# Per- and Polyfluoroalkyl Substances in Fish: Global Occurrence, Bioaccumulation, Analytical Approaches, and Human Exposure Risks—A Review

**DOI:** 10.3390/toxics14040336

**Published:** 2026-04-16

**Authors:** Ines Varga, Nina Bilandžić, Jelena Kaurinović, Andrea Gross Bošković, Tomislav Klapec

**Affiliations:** 1 Laboratory for Residue Control, Department of Veterinary Public Health, Croatian Veterinary Institute, Savska Cesta 143, 10000 Zagreb, Croatia; varga@veinst.hr (I.V.); kaurinovic@veinst.hr (J.K.); 2 Croatian Agency for Agriculture and Food, Kardinala Alojzija Stepinca 17, 31000 Osijek, Croatia; andrea.gross-boskovic@hapih.hr; 3 Department of Applied Chemistry and Ecology, Faculty of Food Technology, Josip Juraj Strossmayer University in Osijek, F. Kuhača 18, 31000 Osijek, Croatia; tomi@ptfos.hr

**Keywords:** per- and polyfluoroalkyl substances (PFAS), persistent pollutants, aquatic environments, fish contamination, bioaccumulation, human dietary exposure, analytical methods

## Abstract

Per- and polyfluoroalkyl substances (PFAS) are highly stable and persistent environmental contaminants. Their exceptional chemical stability prevents natural breakdown, leading to global distribution and bioaccumulation in aquatic organisms. Long-chain PFAS, such as perfluorooctane sulfonic acid (PFOS), tend to accumulate in the liver, kidneys, and muscle tissues, whereas short-chain PFAS remain largely dissolved in water and show lower accumulation. Freshwater fish generally contain higher PFAS levels than marine fish, with concentrations varying according to species, habitat, trophic level, contamination site, and other factors. Human exposure primarily occurs through the consumption of contaminated fish and seafood, as well as through drinking water, inhalation, and skin contact. Such exposure is associated with immunosuppression, high cholesterol, hormonal disruption, cancer, and other health risks. Regulatory limits exist for four PFAS compounds, while many others, including emerging compounds, remain unregulated. This review synthesizes the current knowledge on the global distribution of PFAS across various fish species, analytical approaches including sample preparation (e.g., SPE, QuEChERS) and instrumental techniques (e.g., LC-MS/MS, HRMS), human dietary exposure, and the related health risks. By integrating environmental distribution, bioaccumulation, analytical challenges, and health issues, this review provides an up-to-date perspective on PFAS in fish and emphasizes the need for ongoing monitoring and stricter regulatory frameworks to ensure food safety and protect both human health and ecosystems.

## 1. Introduction

Per- and polyfluoroalkyl substances (PFAS) are emerging pollutants with highly stable carbon-fluorine (C–F) bonds, among the strongest in organic chemistry [1,2,3]. These synthetic compounds are known as “forever chemicals” and include more than 15,000 compounds [4,5]. They consist of a hydrophobic carbon chain that is either fully or partially fluorinated and a terminal hydrophilic functional group such as sulfonate, phosphonate, sulfonamide, carboxylate, or alcohol [6,7]. Based on chain length, PFAS are classified as long-chain and short-chain compounds [8].

Early forms of PFAS, such as polychlorotrifluoroethylene (PCTFE) and polytetrafluoroethylene (PTFE, commonly known as Teflon^®^), were developed in the 1930s [9,10]. Since the 1940s, PFAS have been extensively used across a wide range of products, including firefighting foams, pesticides, semiconductors, non-stick cookware, food-packaging materials, paints, textiles, and cosmetics [3,11,12]. As a result, PFAS are found worldwide in water, biota, and air and can be transported to distant areas, including the Arctic and Antarctic, via atmospheric and ocean currents [11,13].

Two well-known PFAS, perfluorooctanoic acid (PFOA) and perfluorooctane sulfonic acid (PFOS), are toxic to humans and animals due to multiple toxic effects [13]. PFOS, PFOA, and perfluorohexane sulfonic acid (PFHxS), along with their salts and related compounds, were added to the Annexes of the Stockholm Convention on Persistent Organic Pollutants (POPs) in 2009, 2019, and 2022, respectively [14,15]. Following this listing, the use of these compounds has been regulated [13].

Due to their extensive application and strong carbon-fluorine bonds, PFAS are highly resistant to degradation, leading to their persistence in aquatic ecosystems. In the environment, PFAS bioaccumulate in various species, including marine mammals, birds, invertebrates, and fish. Organisms at higher trophic levels typically exhibit elevated concentrations, reflecting trophic transfer throughout the food web. This biomagnification underscores the potential risk to top predators, including humans [16,17]. As a widely consumed food, fish provides essential nutrients, and regular consumption is associated with various health benefits [18]. However, it also represents a significant pathway of human exposure to PFAS [19].

PFAS have been widely studied in environmental matrices and animal-derived foods. Reported PFAS concentrations in fish vary depending on sample preparation, analytical techniques, instrument sensitivity, selection of target compounds, fish species, and reporting units. Research continues to expand the understanding of PFAS occurrence, bioaccumulation, human dietary exposure, and associated health effects.

While numerous reviews have covered multiple matrices, this overview specifically focuses on PFAS in fish and summarizes: (1) sources, behavior, and bioaccumulation; (2) global occurrence across fish species; (3) extraction and analytical methodologies; (4) human exposure through fish consumption and potential health effects; (5) emerging PFAS. The literature was collected from major scientific databases, including Web of Science Core Collection, PubMed, ScienceDirect, and MDPI, focusing primarily on studies published in the past five years, along with key earlier studies. Both original research articles and reviews were selected using keywords related to aquatic organisms, analytical techniques, target compounds, occurrence, and human exposure. By integrating these aspects, this article provides an up-to-date overview of PFAS in fish, highlights current knowledge and analytical challenges, and emphasizes the need for continued research and monitoring.

## 2. Source, Transport, and Fate of PFAS in Aquatic Systems

PFAS are continuously released into aquatic systems, primarily through the manufacture, use, and disposal of various products via both direct and indirect pathways. Direct inputs include industrial production and discharge, use and disposal of consumer products, aqueous film-forming foams, and emissions from specific industrial sectors such as electronics, paper, and semiconductor production. Indirect inputs primarily result from the transformation and release of precursor compounds, ongoing impacts of past emissions, secondary releases from wastewater treatment plants (WWTP), and atmospheric deposition [20]. WWTP, both industrial and municipal, represent major sources of PFAS worldwide. For example, in Switzerland, sewage sludge from 45 WWTP contained various long-chain PFAS at concentrations up to 75 µg/kg. During these processes, PFAS can be transformed into more persistent compounds, particularly short-chain C4–C7 perfluoroalkyl carboxylic acids (PFCAs), which dominate the effluents. Monitoring studies report that perfluorobutanoic acid (PFBA) and perfluorobutane sulfonic acid (PFBS) account for more than 50% of detected short-chain PFAS [21].

PFAS have been detected at higher levels in freshwater systems, including rivers and lakes. Levels are particularly elevated near industrial zones, urban areas, fire-training sites, and WWTP compared to background areas. In some lakes and rivers, they range from a few thousand ng/L to tens of thousands of ng/L. The most frequently detected compounds are perfluoroalkane sulfonic acids (PFSAs) and PFCAs such as PFOS, PFOA, PFHxS, and perfluorononanoic acid (PFNA) [20]. Over 90% of European river samples contain one or more PFAS compounds, highlighting their widespread presence in drinking water [22]. Global monitoring indicates regional variations in PFAS contamination. Median PFOS concentrations were 5.11 µg/L in Europe, 7.40 µg/L in America, and highest in the Asia-Pacific region at 8.46 µg/L. The Asia-Pacific region also exhibited the highest PFOA concentration (4.22 µg/L), likely due to slower regulatory implementation, ongoing industrial production, and regional lifestyle factors [23]. In some United States water sources, concentrations reach up to 10 mg/L, likely due to proximity to firefighting training sites, manufacturing facilities, and extensive monitoring. In Europe, especially in Spain, the Netherlands, and Belgium, concentrations have exceeded 1 mg/L, reaching over 2 mg/L in Belgium, while in China they are up to 5 mg/L. Considerably lower levels have been reported in Canadian waters (0.0001–0.010 mg/L). A global assessment of 45,000 drinking and groundwater samples reported the highest proportion of total PFAS concentrations (20 compounds) in North America (44.3%), followed by Europe and Australia (both 24.6%), with lower percentages in Asia (6%), South America and Africa (both 0.3%) [21].

PFAS persist in aquatic ecosystems due to their strong bonds, which also enable their transfer through food webs. Their environmental behavior depends on chemical properties, environmental conditions, and electrostatic interactions with particulate organic matter. Long-chain compounds tend to persist longer and bioaccumulate more readily in fish. In contrast, short-chain analogues are more mobile, hydrophilic, water-soluble, and challenging to eliminate from water [21,24]. Older fish may accumulate higher concentrations due to prolonged exposure, whereas short-chain PFAS rarely do so unless water concentrations are elevated [25]. Additionally, PFSAs are more prone to bioaccumulate than PFCAs [11]. Both branched and linear PFAS isomers occur in the environment, but they behave differently. Linear PFOS and PFOA have a higher affinity for serum albumin, while branched forms show different degradation patterns and toxic impacts on humans and animals [26,27]. PFAS not only bioaccumulate in individual organisms but also biomagnify through the food web, reaching species at higher trophic levels [20,26].

PFAS precursors are metabolized by enzymatic processes in fish into persistent transformation products, such as PFSA and PFCA [11]. For example, compounds such as perfluoroalkane sulfonamides (FASA), sulfonamidoacetic acids (FASAA), and sulfonamidoethanols (FASE) can be transformed into PFOS, a predominant compound in marine wildlife [16]. The biotransformation of precursors increases with trophic level, from invertebrates to fish. This process is species-specific and involves specific proteins, resulting in variable contamination profiles [28].

While PFAS bioaccumulate and transform within organisms, their environmental persistence also depends on water–sediment interactions. These interactions are influenced by environmental factors (e.g., salinity, pH, dissolved organic matter, and hydrodynamic conditions) and the structural properties of PFAS (e.g., chain length, functional groups, and chemical stability). Higher pH can reduce electrostatic sorption and enhance PFAS mobility. In contrast, increased salinity and ionic strength promote sorption to sediments or suspended particles. Fine sediment particles with higher organic carbon content show greater sorption capacity for long-chain PFAS. Conversely, dissolved organic matter can compete for sorption sites, reducing PFAS retention (e.g., PFOS and PFOA) and releasing them back into the environment. Sulfonic groups (–SO_3_H) tend to bind to sediments due to higher hydrophobicity and larger molecular volume, whereas carboxylic groups (–COOH) remain more prevalent in water. This sorption also enhances complexation with organic matter in sediments [20]. Higher salinity and PFAS chain length increase build-up in suspended particles and sediments, leading to bioaccumulation in benthic organisms. Under these conditions, fish can rapidly accumulate long-chain PFAS, often within hours or days [29]. Consequently, sediments can act as reservoirs and sources of PFAS, exposing organisms that live in, feed on, or interact with them. This also facilitates PFAS transfer, bioaccumulation, and biomagnification across aquatic species and higher trophic levels, including top predators, ultimately increasing fish exposure and adverse effects [30,31,32,33]. A case study in Kent Lake, USA, by Endicott et al. [34] supports these findings, showing that PFOS and 6:2 fluorotelomer sulfonic acid (6:2 FTS) in sediments contribute to PFAS exposure in aquatic organisms, as indicated by contamination patterns in sediment, water, and biota, along with bioaccumulation factors and partition coefficients.

The industry has adopted several short-chain PFAS as alternatives to PFOS and PFOA, including 4,8-dioxa-3H-perfluorononanoic acid (DONA), hexafluoropropylene oxide-dimer acid (HFPO-DA or GenX), and 6:2 chlorinated polyfluorinated ether sulfonate (6:2 Cl-PFESA) [25,35,36,37]. These emerging compounds offer higher solubility, faster elimination, and potentially lower toxicity and bioaccumulation. Nonetheless, they persist and occur in biotic and abiotic environments. Further research is required on their environmental fate and toxicological effects, particularly in edible marine species, to better assess risks to the environment and human health [36].

In conclusion, regional differences in PFAS concentrations reflect variations in industrial emissions, WWTP, and regulatory enforcement, highlighting the need for stricter and more uniform regulations. Bioaccumulation in fish depends on trophic position, chain length, functional groups, sediment interactions, and other environmental conditions. Long-chain PFAS are well documented, but the results vary by species, sampling site, analytical method, and local environmental conditions, highlighting knowledge gaps. Emerging short-chain PFAS remain poorly understood, requiring further investigations of their environmental behavior and potential toxic effects on aquatic organisms and humans.

## 3. Global Distribution of PFAS in Fish Tissues

Research across multiple regions has shown the presence of PFAS in both freshwater and marine fish. For example, fish from Spain (Mediterranean Sea) exhibited elevated concentrations of long-chain PFAS, including PFNA, perfluoroundecanoic acid (PFUnDA), and perfluorododecanoic acid (PFDoDA), whereas PFOS was the predominant compound in fish from the United Kingdom (Atlantic Ocean). These variations likely reflect both past and ongoing emissions from industrial, wastewater, and atmospheric sources in the Mediterranean Sea. Ultra-short-chain PFAS, including trifluoroacetic acid (TFA) and trifluoromethanesulfonic acid (TFSA), were also frequently detected in the UK fish, while PFOA concentrations generally increased with fish size [38]. A similar pattern was observed across other European regions. In the Baltic Sea, PFOS was detected in all fish samples and in over 80% of freshwater fish, with smelt exhibiting the highest levels. Long-chain PFCAs, including PFNA, perfluorodecanoic acid (PFDA), and PFUnDA, were more prevalent in freshwater species, while PFOA was detected only in Baltic herring and smelt [39]. In the Sava River Basin, PFOS occurred in 80% of samples, with PFOA and perfluorohexanoic acid (PFHxA) present in 70% of freshwater fish, at concentrations of 5.9–17.0 ng/g, 2.5–8.0 ng/g, and 0.5–33.2 ng/g wet weight (ww), respectively [40]. In contrast, Mediterranean marine fish, such as Atlantic bluefin tuna (*Thunnus thynnus*), contained lower PFOS concentrations (0.4–1.88 ng/g), whereas PFOA was not detected [41]. Overall, freshwater fish typically accumulate higher PFAS levels than marine species, likely reflecting upstream industrial activities, which pose a greater risk to consumers [42,43].

In East Asia, PFOS was present in all fish samples from Chinese markets. Long-chain PFCAs, including PFOA, PFNA, PFDA, PFUnDA, PFDoDA, and perfluorotridecanoic acid (PFTrDA), as well as the emerging compound 6:2 Cl-PFESA, were detected in ~80% of samples. In contrast, PFHxA and PFHxS were detected less frequently (<60%) [44]. In South Korea, total PFAS concentrations varied among regions and species. For example, Japanese amberjack (*Seriola quinqueradiata*) in samples from Busan exhibited higher concentrations (3.04 ng/g ww) compared to Masan (1.87 ng/g ww), while Korean rockfish (*Sebastes schlegelii*) showed higher levels in samples from Masan (1.31 ng/g ww) than from Busan (0.99 ng/g ww). Similarly, grey mullet (*Mugil cephalus*) showed regional variation across Masan, Busan, and Gwangyang (1.03, 1.93, and 2.63 ng/g ww, respectively). These variations likely reflect local environmental conditions as well as industrial and shipping activities [45].

PFAS have also been detected in fish from remote areas, including Antarctica, Patagonia, and northern Chile. In Antarctica, the sodium salt of DONA (NaDONA) and HFPO-DA were detected for the first time, while PFBS, perfluoropentane sulfonic acid (PFPeS), and perfluoroheptane sulfonic acid (PFHpS) were the dominant compounds [46].

These findings indicate the presence of PFAS in both freshwater and marine fish, with regional differences in dominant compounds. Long-chain compounds are prevalent in the Mediterranean Sea, while PFOS dominates in Atlantic and Baltic fish. PFAS levels are generally higher in freshwater fish than in marine species. They have also been detected in remote regions, including Antarctica, highlighting their widespread environmental persistence and global distribution.

In addition to the studies discussed above, Table 1 presents additional selected PFAS data from various fish species across different regions.

Across regions and species, PFOS was the most frequently detected compound, while long-chain PFCAs such as PFUnDA, PFDA, PFDoDA, and PFNA were also common. Short-chain PFAS were generally less prevalent. In Swiss lakes, perch exhibited the highest PFAS diversity, followed by brown trout, rainbow trout, and common carp. This pattern is related to the carnivorous diet of perch, which includes macroinvertebrates, zooplankton, and other fish, leading to greater bioaccumulation [47]. PFOS concentrations were highest in carnivorous species such as brown trout (157.00 µg/kg) and lower in omnivorous species such as common carp (6.77 µg/kg) [47]. This is in accordance with the literature and reflects variations across trophic groups, with generally higher accumulation in carnivorous fish compared to omnivorous or herbivorous species [30,45].

According to Kuc et al. [27], PFBA, PFNA, and PFOS exhibited greater variability in fish tissues across Baltic Sea fisheries compared to PFBS, perfluoroheptanoic acid (PFHpA), and PFHxS, likely due to bioaccumulation, biodilution, and biomagnification [27]. In the Baltic Sea and Finnish waters, PFOS was predominant in European perch and herring, alongside other long-chain PFAS [27,48]. Perch from a point source-polluted site exhibited higher PFOS concentrations than herring from diffuse source-polluted sites [48], suggesting that sampling sites influence PFAS accumulation.

Total PFAS concentrations varied between the Baltic Sea [27] and the Saudi Arabian Red Sea [52], with slightly higher concentrations in the Red Sea. ∑PFAS ranged from 1.9 to 4.8 µg/kg in the Baltic Sea and from 3.89 to 7.63 µg/kg (mean) in the Red Sea [27,52]. These differences may be influenced by factors such as species, habitat, diet, trophic level, and local environmental contamination.

In East Canyon Creek (Utah), PFOS was detected at the highest levels in both brown trout (higher trophic level) and mottled sculpin (lower trophic level), with only minor differences between species. Total PFAS levels followed a similar pattern, and no clear impact of wastewater discharge or significant biomagnification was observed [49]. In Sub-Saharan Africa, notably higher PFAS concentrations in smoked fish from Mali suggest local contamination sources, likely associated with pesticide use [50]. In the Veneto Region (Italy), freshwater fish from a fluorochemical contaminated area exhibited variable concentrations among species, with Italian barbel being the most contaminated, followed by chub, wels catfish, and carp, likely related to feeding behavior and habitat [54].

PFAS accumulation also differs among tissues. For example, Clements et al. [53] reported that PFAS levels in the liver were 1.13–350.1 times higher than in muscle. This is associated with the strong binding of PFAS to proteins abundant in hepatocytes such as serum albumin and fatty acid-binding proteins [16]. The distribution pattern across fish tissues generally follows: liver > kidney > muscle [30]. Overall, these studies analyzed only 10–39 compounds out of many known, which may underestimate the total PFAS levels in fish.

According to the literature, PFAS accumulation in fish is influenced by multiple factors, including species, age, size, habitat, diet, lipid content, isomer composition, fillet type, and fish origin [27,30,43,45,55,56]. For instance, skinless fillets from farmed fish contained lower PFOA and PFOS levels compared to wild fish. Similarly, in wild freshwater fish, PFOS levels were lower in skinless fillets than in fillets with skin from the same species [55]. Overall, wild fish exhibit higher PFAS levels due to greater environmental exposure [57]. Lipid content influences the bioaccumulation of lipophilic compounds in fish, which also depends on chain length and functional group [43,55]. PFAS isomer composition also varies among fish species and habitats. For instance, lake fish generally contain a lower proportion of linear PFOS than river fish, while PFOA shows no significant differences. Branched isomers can account for a significant portion of total PFAS, although the reasons are unclear [56]. Finally, fish size, including both weight and length, is an additional factor influencing PFAS concentrations [45].

## 4. Sample Preparation and Quantification of PFAS in Fish

Fish represent a complex matrix composed of proteins, fats, and other components, which may interfere with analytical determination [58]. In such matrices, matrix effects (MEs) are common and occur when analytes are eluted alongside other molecules. These effects can affect critical analytical parameters, including linearity, precision, accuracy, limit of detection (LOD), and limit of quantification (LOQ) [59,60]. To minimize MEs, strategies such as internal standards, matrix-matched calibration, and optimized extraction and clean-up are frequently employed. Without these measures, signal enhancement or suppression can cause deviations in measured values [58,59,61]. MEs are typically evaluated by comparing the analytical response of matrix-matched calibration with standards prepared in solvent. This comparison indicates the influence of interferences on the analytical signal and overall method efficiency [58]. Some studies provide examples of these evaluations. For instance, Koloka et al. [58] applied QuEChERS-sonication extraction (Quick, Easy, Cheap, Effective, Rugged, and Safe) to determine 14 PFAS in fish. They observed low MEs (−19.5% to 7.8%) for eight compounds and medium MEs (−39.4% to 20.7%) for six compounds. Similarly, Chiesa et al. [19] evaluated MEs for 17 PFAS in fish and reported a low impact (<20%) using solvent extraction followed by solid-phase extraction (SPE), confirming that careful sample preparation reduces matrix interferences. Despite these strategies, the optimization of extraction and purification remains crucial to enhance selectivity and sensitivity, considering the chemical and physical properties of the target analytes [61,62].

Various procedures have been applied to extract PFAS from fish samples. Sample preparation often employs QuEChERS [63,64], a rapid and simple approach employing solvent extraction (e.g., acetonitrile) with dispersive solid-phase extraction (dSPE) [61]. This procedure usually includes salts (NaCl and MgSO_4_) [63] and sorbents (C18, EMR-Lipid, Z-Sep, Envi-Carb, and PSA), used individually or in combination to remove lipids and polar matrix components such as fatty acids and sugars [59]. The QuEChERSER method (Quick, Easy, Cheap, Effective, Rugged, Safe, Efficient, and Robust) has also been introduced [65]. SPE is commonly carried out with weak anion exchange (WAX) cartridges (e.g., Strata-XL-AW) [66] or weak anion exchange/graphitized carbon black (WAX/GCB) cartridges (e.g., StrataVR PFAS WAX/GCB) [67]. Other extraction techniques include ion-pairing extraction [68], accelerated solvent extraction (ASE) [69], ultrasound-assisted extraction [27], and alkaline extraction [35].

For instrumental analysis, liquid chromatography (LC) or ultra-high performance liquid chromatography (UHPLC) [68] is often coupled with tandem mass spectrometry (MS/MS) [65,67,68,69] or high-resolution mass spectrometry (HRMS) [63,64], such as quadrupole time-of-flight tandem mass spectrometry (QTOF-MS/MS) [68]. LC-HRMS allows targeted, non-targeted, and suspect analysis [58], enabling identification of PFAS precursors and potential structures of unknown compounds, without providing quantitative data [68]. Recently, liquid chromatography-ion mobility spectrometry-mass spectrometry (LC-IMS-MS) has also been applied [70]. To detect and quantify PFAS, negative electrospray ionization (ESI–) is widely applied, particularly for acidic compounds, including PFCAs, PFSAs, fluorotelomer sulfonates, and sulfonamidoacetic acids, as well as some neutral compounds such as perfluorooctanesulfonamide (FOSA) [40,71]. The use of a delay column is recommended to reduce potential contamination from the LC system and mobile phases [72].

While several extraction, clean-up, and analytical methods are used, the most commonly applied techniques are compared. SPE includes several steps and large solvent volumes, making it time-consuming. In contrast, QuEChERS is simpler, faster, easier to handle, more efficient, and uses less solvent [73]. Gallocchio et al. [61] compared SPE (WAX) and QuEChERS for PFAS extraction in various matrices, including fish, bovine muscle, eggs, and milk. SPE provided clean extracts with satisfactory recoveries (80–120%) and repeatability (CV < 20%), but required two days to process ~20 samples, whereas QuEChERS achieved comparable results with a shorter preparation time (~30 samples in one day). No significant MEs were observed, enabling the use of standard solution calibration curves instead of matrix-matched calibration. These findings suggest that QuEChERS can effectively replace SPE for routine analysis, providing satisfactory validation results while facilitating laboratory workflow and saving time.

LC-MS/MS is widely applied for targeted PFAS analysis, where compounds of interest are known and reference standards are available. However, reference standards for many PFAS remain limited. In contrast, HRMS offers full-scan acquisition and accurate mass measurement, enabling targeted, suspect, and non-targeted screening. Suspect screening combines targeted and non-targeted approaches, allowing identification of “known unknown” analytes. Non-targeted approaches are useful for identifying unknown compounds and monitoring transformation products. HRMS can be easily coupled with LC, GC, or ion mobility (IM), enabling detection of a larger number of PFAS compared with targeted analysis. The main limitations include high acquisition and maintenance costs, the need for specialized technical expertise, large datasets, and slower processing compared with LC-MS/MS. However, recent developments allow faster and more efficient data analysis [73,74].

Table 2 summarizes relevant parameters, including the number of PFAS, sample amount, extraction method, analytical technique, chromatographic column, mobile phase, recovery, and validation limits in fish.

According to Table 2, the number of targeted PFAS analytes ranges from 10 to 47, while sample amounts vary from 0.1 g to 10 g, reflecting differences among the studies. SPE is the most commonly used extraction technique, followed by QuEChERS, although some studies employ methods such as alkaline extraction with ultrasonication or ultrasound-assisted extraction. Most studies employ targeted LC-MS/MS analysis using C18 columns with varying dimensions and particle sizes (1.7–5 µm). Mobile phases generally consist of ammonium acetate or ammonium formate in water combined with an organic phase (methanol or acetonitrile). Detection limits (LOD/MDL) range from 0.002 ng/g [38] to 20 ng/g [47], while quantification limits (LOQ/LLOQ/MQL) range from 0.005 ng/g [75] to 50 ng/g [47]. In conclusion, careful selection and optimization of sample preparation and instrumental parameters are crucial to achieve reliable analytical performance that meets regulatory requirements.

Despite advances in extraction and analytical techniques, several challenges remain. These include matrix effects, interferences, ion suppression or enhancement, limited reference and internal standards, limited certified reference materials, high LOQs for certain matrices, high analytical costs, the lack of a single analytical method capable of covering all PFAS classes, and potential contamination from laboratory equipment [74,77,78]. These challenges highlight the need for further development and standardization, especially for unknown and emerging PFAS. Continuous improvement of sample preparation and analytical approaches is crucial to ensure reliable and accurate detection and quantification of PFAS in complex matrices.

## 5. Human Dietary Exposure and Food Safety

The main sources of human exposure to PFAS are diet and drinking water, inhalation and dermal absorption [79]. Fish, particularly predatory species and shellfish, often exhibit higher PFAS concentrations due to accumulation within aquatic food webs, which can lead to biomagnification across trophic levels. PFAS bioaccumulation in fish is largely driven by protein binding, particularly to serum proteins, and generally increases with chain length [22]. Higher bioaccumulation factors (BAFs) suggest stronger bioaccumulation potential [29]. For example, BAFs from water to Baltic herring increased from 3.3 to 4.1 for PFSAs (C6–C8) and from 2.0 to 5.3 for PFCAs (C6–C10) [22]. In addition to bioaccumulation, PFAS can biomagnify, as demonstrated by studies in North America, China, and Europe. Trophic magnification factors (TMFs) indicate that PFOS commonly biomagnifies in the food web (TMFs: 0.8–20), while other long-chain carboxylates exhibit significant variability. Geographic location, food web length, sample tissue, sampling methodology, and proximity to PFAS inputs contribute to these differences. Furthermore, PFAS precursors can accumulate and biotransform into more persistent compounds at higher trophic levels, leading to elevated concentrations in predatory fish commonly consumed by humans [80]. Consequently, fish serve as valuable indicators of environmental contamination [22].

Since humans consume fish and seafood, PFAS accumulation in aquatic organisms directly affects human exposure. In the human bloodstream, PFAS primarily bind to serum proteins, increasing their solubility and stability. They also interact with transport proteins in the liver, kidneys, and intestines, promoting bioaccumulation. This effect is stronger for long-chain PFAS, with half-lives of several years, whereas short-chain PFAS are eliminated within several days to about a month [22,31]. Exposure to PFAS has been linked to various health effects. Metabolic impacts include disrupting fat and carbohydrate metabolism, elevating cholesterol, and increasing the risk of cardiovascular disease. Hormonal effects involve increased serum alanine transaminase (ALT) levels [47] and reduced thyroxine (T4) production, particularly for certain long-chain PFAS. Sulfonic compounds generally pose a higher risk to thyroid homeostasis than carboxylic compounds [81]. Additionally, PFOA and PFOS may contribute to infertility and affect testosterone and other sex hormone levels [13]. PFAS can cross the placenta, leading to prenatal exposure, with risks varying by compound [1]. Higher levels in maternal serum have been linked to larger head size and higher body mass index (BMI) in children, potentially increasing obesity risk later in life [82]. They also exhibit carcinogenic potential and have been associated with breast, kidney, lung, and testicular cancers [13]. According to the International Agency for Research on Cancer (IARC), PFOA is classified as possibly carcinogenic to humans. Additionally, sulfonic acid derivatives, including sulfonamides and sulfonates, are considered more toxic and potentially carcinogenic. Human immune responses may be suppressed by PFAS exposure, as controlled laboratory studies reveal that these compounds can alter cytokine expression in immune cells [1]. Finally, PFAS have been found in human blood, hair, and breast milk, confirming their systemic distribution [79].

While these effects are primarily associated with legacy PFAS, emerging PFAS have been shown to pose similar or even greater risks to human health. For example, GenX exhibits greater toxicity to thyroid cells than PFOA and is linked to immune, developmental, reproductive, and carcinogenic effects. Similarly, 6:2 Cl-PFESA can cause liver damage and disrupt endocrine function in mice, and has also been associated with liver cancer in humans. Toxic effects have likewise been observed for the ammonium salt of DONA (ADONA) [37].

Maximum levels (MLs) for the four PFAS in food, including their sum, have been established under Commission Regulation (EU) 2023/915. Depending on the fish species, these levels range from 2.0 to 35 μg/kg for PFOS, 0.20 to 8.0 μg/kg for PFOA, 0.50 to 8.0 μg/kg for PFNA, and 0.2 to 1.5 μg/kg for PFHxS, with the sum of the four PFAS (∑4PFAS) ranging from 2.0 to 45 μg/kg [83]. Recent notifications from the Rapid Alert System for Food and Feed (RASFF) indicate the occurrence of PFAS in various fish, with measured concentrations as follows: mackerel (PFAS: 5.05 µg/kg), carp (PFOS: 6.13 µg/kg and 3.2 µg/kg), horse mackerel (PFAS: 50.487 ± 4.372 µg/kg), turbot (PFOS: 2.50 µg/kg), and common sole (PFOS: 3.6 µg/kg; PFNA: 1.5 µg/kg). Among the quantified compounds, PFOS was most commonly observed. These occurrences were reported in 2024 and 2025 [84].

To limit potential health risks, the European Food Safety Authority (EFSA) has defined a tolerable weekly intake (TWI) of 4.4 ng/kg body weight (bw)/week for the total intake of PFNA, PFOA, PFHxS, and PFOS [13]. Actual fish consumption often leads to TWI exceedances. For example, consuming 200 g of Baltic Sea fish per week exceeds the TWI for 10 of 13 species studied, with smelt exhibiting the highest levels. PFAS intake from other sources is not included [39]. Similarly, TWI exceedances were observed in most fish from Swiss lakes, including 95% of whitefish, 100% of common chub, 55% of rainbow trout, 50% of brown trout, and 36% of perch [47].

This trend is further confirmed by a study of five Baltic fish species (trout, salmon, cod, herring, and sprat), showing that children are at the highest risk of exceeding the EFSA TWI, with intake ranging from 3.20 to 25.13 ng/kg bw (2–6 times the TWI) depending on portion size, while adults generally remain below the TWI (1.06–8.30 ng/kg bw). Among all analyzed species, sprat showed the highest ∑4PFAS intake. PFAS levels in fish can increase significantly with grilling or frying, whereas cooking has no effect, which should be considered in risk assessments [85].

Similar exceedance was observed in a study from the United Kingdom (UK) and Spain. Spanish consumers had higher exposure (24.62 ng/kg bw/week) compared with British consumers (10.71 ng/kg bw/week), primarily due to elevated PFOS and PFNA levels. Mean PFNA intake from Spanish fish (2.06 ng/kg) was about 35 times higher than from UK fish (0.06 ng/kg). At median intake (P50), Spanish consumers (3.04 ng/kg) were closer to the EFSA limits than British consumers (2.76 ng/kg) [38].

According to Rampazzo et al. [86], toddlers exhibit the highest estimated weekly intake (EWI) from fish and seafood consumption (22.62–25.93 ng/kg bw/week), exceeding the TWI by 514–589%. PFOS is the main contributor (up to 354% of the TWI), followed by PFNA (58%), PFDA (44–110%), PFOA (31%), and PFUnDA (17%). In other age groups (adolescents, adults, and the elderly), EWIs are lower but still exceed recommended limits (12.06–14.58 ng/kg bw/week; 274–331% of the TWI), with PFOS again predominating (175–199%), followed by PFNA (31–33%), PFOA (29–33%), PFDA (23–62%), and smaller contributions from PFUnDA and PFTrDA.

Importantly, conventional risk assessment often focuses on individual PFAS such as PFOA and PFOS, while human exposure typically occurs as complex mixtures from both environmental and dietary sources. This highlights the need for novel methods to assess total toxicity more effectively [86], rather than focusing solely on individual compounds, particularly in vulnerable populations. Since fish and seafood consumption can lead to exceedances of safety limits in certain population groups and pose long-term health risks, regular monitoring remains crucial to protect human health [16,32,47].

## 6. Conclusions

PFAS are widespread and persistent pollutants in aquatic ecosystems, including freshwater and marine fish. Their bioaccumulation is influenced by habitat, protein content, fish size, trophic level, pollution sources, and other factors. PFAS primarily accumulate in the liver, followed by the kidneys and muscle tissue, with carnivorous and freshwater species generally exhibiting higher levels. Human exposure occurs mainly through fish consumption, particularly from predatory species, and in certain regions, intake of PFAS-contaminated fish may exceed the TWI, representing a potential health risk, especially for vulnerable populations.

To protect humans, regulatory limits have been established for four PFAS (PFOS, PFOA, PFHxS, and PFNA), but many other compounds remain unregulated. Advances in analytical methods have improved detection and quantification, yet challenges remain, particularly for unknown or emerging PFAS. Further research should expand the range of PFAS analytes in regulatory frameworks and monitoring programs, develop multi-method approaches for broader detection, and standardize analytical methods to ensure comparability across studies. Emphasis should be placed on LC-HRMS, including suspect and non-target screening, to detect unknown PFAS and their degradation products. Investigating emerging PFAS and their environmental behavior, along with ongoing monitoring, is crucial for establishing stricter regulations, assessing risks, and providing dietary recommendations to protect consumers.

## Figures and Tables

**Table 1:** Global occurrence of PFAS in fish species.

Region	Fish Species	Tissue	Number of PFAS	Concentration Range	Key Findings	**Reference**
Swiss Lakes	Whitefish (*Coregonus wartmanni*), common carp (*Cyprinus carpio*), perch (*Perca fluviatilis*), rainbow trout (*Oncorhynchus mykiss*), common chub (*Squalius cephalus*), and brown trout (*Salmo trutta*)	Fillet	15 PFAS	PFOS: 0.69–109.90 µg/kg (whitefish), 0.10–6.77 µg/kg (common carp), 0.13–19.40 µg/kg (rainbow trout), 0.01–41.50 µg/kg (perch), 0.01–157.00 µg/kg (brown trout), 1.16–21.30 µg/kg (common chub)	PFOS was dominant across all species, with highest accumulation in brown trout. Perch showed greatest PFAS diversity (all 15 PFAS detected), followed by brown trout, rainbow trout, and common carp.	[47]
Baltic Sea	Flatfish (*Platichthys flesus*), herring (*Clupea harengus*), and perch (*Perca fluviatilis*)	Muscle	10 PFAS	∑PFAS = 1.9–4.8 µg/kg	PFOS was dominant in most samples; PFBA, PFNA, PFDA, and PFOS detected in all samples above LOQ.	[27]
Finnish inland, coastal open sea waters	Baltic herring (*Clupea harengus membras*) and European perch (*Perca fluviatilis*)	Fillet	23 PFAS	PFOS: perch mean 3.4 µg/kg (max. 18 µg/kg), herring mean 0.49 µg/kg (max. 1.6 µg/kg) ∑PFAS: perch 0.98–31 μg/kg, herring 0.22–2.4 μg/kg	PFOS detected in 100% of samples. In perch, besides PFOS, other long-chain PFAS (PFUnDA, PFTrDA, PFDA, PFNA and PFDoDA) were also detected in 100% of samples. Perch contained 17 PFAS, while Baltic herring contained 10 PFAS.	[48]
East Canyon Creek, Utah, USA	Mottled sculpin (*Cottus bairdii*) and brown trout (*Salmo trutta*)	Fish sample	35 PFAS	∑PFAS: sculpin 0.46–63.9 ng/g, brown trout < LOQ–52.1 ng/g PFOS: sculpin 3.8–46.5 ng/g, brown trout 2.5–38.4 ng/g	PFDA most frequently detected (93%) in brown trout and sculpin samples, followed by FOSA (83% in sculpin and 79% in brown trout). PFOS at highest levels with detection frequency 67% in sculpin and 83% in brown trout.	[49]
Sub-Saharan Africa (Mali, Cameroon, Benin, and Nigeria)	Sea fish, smoked fish, and fresh water fish	Fillet	14 PFAS	PFOS = <0.02–10.44 µg/kg Long-chain PFCAs = 0.01–0.89 µg/kg	PFOS most frequently detected. Highest concentration of PFOS in smoked fish from Mali. Detection rates: PFOS and PFUnDA (89%), PFNA, PFDA and PFDoDA (67%).	[50]
Bahia, Brazil	Mojarra (*Diapterus* sp.), torroto grunt (*Genyatremus luteus*), catfish (*Aspistor luniscutis*), drum (*Stellifer* sp.), madamango sea catfish (*Cathorops spixii*), drum (*Caranx* sp.), barbel drum (*Ctenosciaena gracilicirrhus*), mullet (*Mugil* sp.), fat snook (*Centropomus parallelus*), common snook (*Centropomus undecimalis*), and silver jenny (*Eucinostomus* sp.)	Muscle	20 PFAS	L-PFOS: mean 0.24–1.20 ng/g br-PFOS: mean 0.04–0.24 ng/g ∑PFAS: mean 0.45–1.75 ng/g	PFOS most abundant; long-chain PFAS also detected.	[51]
Saudi Arabian Red Sea	bluefin trevally (*Caranx melampygus*), marbled spinefoot (*Siganus rivulatus*), bonefish (*Albula glossodonta*), bigeye scad (*Selar crumenophthalmus*), doublespotted queenfish (*Scomberoides lysan*), and strongspine silver-biddy (*Gerres longirostris*)	Muscle	23 PFAS	∑PFAS: mean 3.89–7.63 µg/kg	PFOS and PFUnDA dominated in all muscle samples (max. 15.13 and 0.84 µg/kg), followed by PFDA (0.80 µg/kg, 98% samples). Long-chain PFAS in 48% of samples; short-chain PFAS in <25%.	[52]
Cochiti and Abiquiu Reservoirs, Rio Grande, New Mexico	Fish from reservoir: smallmouth bass (*Micropterus dolomieu*), common carp (*Cyprinus carpio*), white crappie (*Pomoxis annularis*), catfish (*Ictalurus punctatus* or *Ictalurus furcatus*), white sucker (*Catostomus commersonii*), northern pike (*Esox Lucius*), and walleye (*Sander vitreus*) Fish from Rio Grande: blue catfish, white sucker, common carp, and channel catfish	Fillet (muscle) and liver	39 PFAS	Muscle: ∑PFAS: mean 2.02 ± 1.81 ng/g, positive detections 0.169–4.22 ng/g PFOS: highest 0.414–4.22 ng/g, (mean: 1.87 ng/g) Liver: PFOS 2.94–146 ng/g (mean: 41 ng/g)	Muscle: PFOS most frequently detected (95%), followed by PFDA and PFUnDA Liver: PFOS predominant, concentrations 1.13–350.1× higher than muscle	[53]
Veneto, Italy	Carp, Italian barbel, wels catfish, channel catfish, rainbow trout, and chub	Fillet	12 PFAS	PFOS: mean 9.23 µg/kg PFUnDA: mean 0.55 µg/kg PFDA: mean 2.87 µg/kg PFDoA: mean 1.51 µg/kg PFOA: mean 0.33 µg/kg	PFOS most abundant (99%), PFUnDA (98%), PFDA (98%), PFDoDA (93%), and PFOA (79%)	[54]

Abbreviations: L-PFOS (linear PFOS); br-PFOS (branched PFOS); ∑PFAS (sum of PFAS); LOQ (limit of quantification); FOSA (perfluorooctanesulfonamide).

**Table 2:** PFAS quantification in fish: sample preparation, analytical techniques, and validation parameters.

Number of PFAS	Sample Amount	Extraction Method	Analytical Technique	Chromatographic Column	Mobile Phase A	Mobile Phase B	Recovery (%)	Validation Limits	**Reference**
21	1 g	SPE (Oasis WAX)	UHPLC-MS/MS	Acquity BEH C18 (2.1 × 100 mm, 1.7 μm)	2 mM ammonium acetate in water	Acetonitrile	79.6–109%	MDL = 2–10 pg/g, except for PFBA (120 pg/g), MQL = 5–30 pg/g, except for PFBA (300 pg/g)	[75]
17	5 g	SPE (Strata PFAS WAX/GCB)	UHPLC-HRMS	Raptor ARC-18 (2.1 × 120 mm, 5 µm)	20 mM ammonium formate in water	Methanol	70–120%	LOD = 15–30 pg/g, LOQ = 50–100 pg/g	[19]
20	5 g	QuEChERS/SPE (WAX)	LC-MS/MS	XBridge BEH C18 (2.1 × 150 mm, 3.5 μm)	5 mM ammonium acetate and 5 mM 1-methylpiperidine in water	Methanol	40–120%	MDL = 11–345 ng/kg (instrument 1), MDL = 12–345 ng/kg (instrument 2)	[66]
15	2 g	Alkaline extraction with ultrasonication	HPLC-MS/MS	Zorbax Eclipse XDB-C8 (3.0 × 100 mm, 3.5 µm)	10 mM ammonium acetate buffer, pH 4.3, in methanol/acetonitrile (90:5:5, *v*/*v*/*v*)	Methanol/acetonitrile (50:50, *v*/*v*)	90–110%	LOD = 0.01–0.02 µg/kg, LOQ = 0.02–0.05 µg/kg	[35]
10	0.1 g	Ultrasound-assisted extraction/SPE (Oasis WAX)	LC-MS/MS	Kinetex XB C18 (2.1 × 100 mm, 2.6 µm)	10 mM ammonium acetate in water	10 mM ammonium acetate in methanol	72–108%	LLOQ = 0.03 µg/kg (PFCAs), LLOQ = 0.1 µg/kg (PFSAs)	[27]
15	10 g	QuEChERS	LC-MS/MS	SB C18 (4.6 × 150 mm, 2.7 µm)	5 mM ammonium acetate in water/methanol (95:5, *v*/*v*)	Water/methanol (5:95, *v*/*v*)	75–115%	LOD = 0.001–0.02 mg/kg, LOQ = 0.007–0.05 mg/kg	[47]
12	5 g	SPE (InertSep WAXFF)	UHPLC-MS/MS	Accura Triart (2.1 × 150 mm, 3 μm)	5 mM ammonium acetate in water	Acetonitrile	80.8–105.3%	LOD = 0.250–0.500 ng/mL, LOQ = 0.500–0.750 ng/mL	[76]
14	2 g	Modified QuEChERS with sonication	LC-HRMS	Hypersil GOLD C18 (2.1 × 100 mm, 1.9 μm)	0.1% formic acid in water	0.1% formic acid in methanol	55.5–113.3%	LOD = 0.01–0.06 ng/g, LOQ = 0.04–0.21 ng/g, CCα = 0.02–0.08 ng/g, CCβ = 0.04–0.13 ng/g	[58]
10	2 g	SPE	LC-QTOF-MS	Atlantis Premier BEH C18 AX (2.1 × 100 mm, 1.7 µm)	2 mM ammonium acetate in water/acetonitrile (95:5, *v*/*v*)	Acetonitrile	–	–	[30]
47	2 g	SPE (Oasis WAX)	UHPLC-HRMS	SeQuant ZIC-HILIC (2.1 × 100 mm, 3.5 μm)	10 mM ammonium formate in water	Methanol	–	LOD = 0.002–0.078 ng/g	[38]

Abbreviations: PFCAs (perfluoroalkyl carboxylic acids); PFSAs (perfluoroalkane sulfonates); LOQ (limit of quantification); LLOQ (lower limit of quantification); MQL (method quantification limit); LOD (limit of detection); MDL (method detection limit); CCα (decision limit); CCβ (detection capability).

## Data Availability

No new data were created or analyzed in this study. Data sharing is not applicable to this article.
